# Drug use patterns among Thai illicit drug injectors amidst increased police presence

**DOI:** 10.1186/1747-597X-4-16

**Published:** 2009-07-21

**Authors:** Dan Werb, Kanna Hayashi, Nadia Fairbairn, Karyn Kaplan, Paisan Suwannawong, Calvin Lai, Thomas Kerr

**Affiliations:** 1 British Columbia Centre for Excellence in HIV/AIDS, St. Paul's Hospital, 608-1081 Burrard Street Vancouver, Canada; 2 School of Population and Public Health, University of British Columbia, Vancouver, Canada; 3 Thai AIDS Treatment Action Group, 18/89 Vipawadee Road, soi 40 Chatuchak, Bangkok 10900, Thailand; 4 Department of Medicine, University of British Columbia, Vancouver, Canada

## Abstract

Thailand has traditionally pursued an aggressive enforcement-based anti-illicit drug policy in an effort to make the country "drug-free." In light of this ongoing approach, we sought to assess impacts of enforcement on drug use behaviors among a cohort of injection drug users (IDU) in Thailand. We examined drug use patterns among IDU participating in a cross-sectional study conducted in Bangkok (*n *= 252). Participants were asked to provide data regarding patterns of drug use in the previous six months, including types of drugs consumed, method of consumption, frequency of use, and weekly income spent on drugs. We also conducted bivariate analyses to identify a possible effect of a reported increase in police presence on measures of drug use and related risk behaviors among study participants. One hundred fifty-five (61.5%) individuals reported injection heroin use and 132 (52.4%) individuals reported injection midazolam use at least daily in the past six months. Additionally, 86 (34.1%) individuals reported at least daily injection Yaba and Ice (i.e., methamphetamine) use. Participants in our study reported high levels of illicit drug use, including the injection of both illicit and licit drugs. In bivariate analyses, no association between increased police presence and drug use behaviors was observed. These findings demonstrate high ongoing rates of drug injecting in Thailand despite reports of increased levels of strict enforcement and enforcement-related violence, and raise questions regarding the merits of this approach.

## Findings

Drug users in Thailand continue to face a variety of harms. In addition to the health risks associated with the consumption of illicit drugs through injection and other means, Thai drug users face stigmatization and an elevated risk of violence as a result of their government's 'hard line' response to illicit drug use [1]. In February 2003, the Thai government implemented a widely-publicized "War on Drugs" aimed at disrupting a burgeoning demand for methamphetamines [2]. The stated goal of this campaign was to make Thailand "drug free" by targeting drug dealers [1,3]. It has been reported that over 2,200 people, not necessarily drug dealers, were killed during its implementation [3]. Despite a massive outcry from human rights groups and a government pledge to treat drug users "as patients, not criminals" [4,5], the reinstitution of the Thai "War on Drugs" was announced in February 2008. At that time, Thailand's interior minister Chalerm Yubamrong publicly stated that the crackdown would continue even if "thousands of people have to die" [6].

Little is known regarding the effect of the Thai War on Drugs on demand for illicit drugs, though recent studies suggest that this campaign may have altered drug use patterns among illicit drug users and reduced consumption of methamphetamine among youth in the short term [7,8]. However, the campaign may have also contributed to a systematic underreporting of illicit drug use and related risk behaviors and may have increased the misuse of diverted licit drugs [7]. This campaign was implemented in response to a massive increase in methamphetamine use among Thais since the mid-1990s, as well as a steady increase in heroin injection that has been linked to the effective eradication of the country's indigenous opium cultivation industry beginning in the 1970s [7]. Research further suggests that the Thai government is continuing to rely on drug crackdowns as a primary response to illicit drug use in the country [9]. We therefore sought to determine the effect of a perceived increase in police presence on drug use patterns among a cohort of Thai injection drug users (IDU) after the announcement of a second Thai "War on Drugs" in April 2008.

The Mitsampan Community Research Project (MSCRP) is a collaborative research project involving the British Columbia Centre for Excellence in HIV/AIDS (Vancouver, Canada), the Mitsampan Harm Reduction Center (Bangkok, Thailand), the Thai AIDS Treatment Action Group (Bangkok, Thailand), and Chulalongkorn University (Bangkok, Thailand). During July-August of 2008, the research partners designed and undertook a cross-sectional study involving IDU recruited from the community through peer-based outreach efforts and word of mouth. Study participants were invited to attend the Mitsampan Harm Reduction Center to participate in the study and all participants provided informed consent and completed an interviewer-administered questionnaire eliciting demographic data as well as information about drug use, health risk behaviors, interactions with police and the criminal justice system, and experiences with health care. All participants were given a stipend of 250 Baht upon completion of the questionnaire. The study has been approved by the Research Ethics Boards of the University of British Columbia and Chulalongkorn University.

For the present analysis, participants were asked to provide detailed data regarding patterns of drug use in the previous six months, including types of drug consumed, method of consumption, frequency of use, and weekly income spent on purchasing drugs. This time period coincided with the implementation of a second "War on Drugs" by the Thai government. We also conducted bivariate analyses in which our dependent variable was a perceived increase in police presence. Specifically, participants were asked the following question: "In the past six months, have you noticed an increase in police presence where you obtain or use drugs?" Independent variables of interest were defined as dichotomous measures of frequent (i.e., ≥ daily *vs. *< daily) injection use of heroin, Yaba or Ice use (i.e., two types of methamphetamines; Yaba translates as "crazy drug" and is produced as a tablet that typically contains methamphetamine and caffeine and is sometimes smoked, while Ice is a smokable form of methamphetamine), illicit (i.e., non-prescribed) methadone use, binge drug use (Yes *vs. *No), involvement in the sex trade (Yes *vs. *No) and involvement in drug treatment (Yes *vs. *No). We also conducted bivariate analyses between our dependent variable of interest (a perceived increase in police presence) and age and gender (male vs. female or transgender). All drug use and behavioral variables refer to the six months prior to the interview. We examined the bivariate associations between each independent variable and a reported increase in police presence using the Pearson *X*^2 ^test. Fisher's exact test was used when one or more of the cells contained values less than or equal to five. Significance was set at the *p *≤ 0.05 level in our analyses. All *p*-values are two-sided.

Two hundred and fifty-two individuals were recruited, including 66 (26.2%) women and 5 (2.0%) transgendered individuals. The median age was 36.5 years. The average weekly income spent on drug use was 423 *baht *(approximately 12 USD). See additional file 1: Table S1 for drug use frequencies reported by our cohort participants. Heroin was the most commonly injected drug, with 155 (61.5%) individuals reporting injection heroin use at least once a day, and 229 (90.9%) individuals reporting at least weekly injection use of heroin in the past six months. The second most commonly injected drug was midazolam, with 132 (52.4%) individuals reporting injection drug use at least once a day in the past six months. Also of note were high levels of Yaba and Ice use, with 86 (34.1%) participants reporting daily injection use and 58 (23.0%) participants reporting daily non-injection use of these drugs. Finally, 73 (29.0%) participants reported using non-injection illicit methadone at least once a day.

In total, 137 (54.4%) participants reported observing an increase in police presence where they purchase or consume drugs in the six months prior to being interviewed. When we performed bivariate analyses to determine the possible association between a reported increase in police presence and drug use patterns, we found no significant associations between our dependent variable and measures of drug use severity or the frequency of binge drug use, involvement in the sex trade, or uptake of addiction treatment among our cohort. Results of bivariate analyses are shown in Table 1.

**Table 1:** Characteristics of Thai injection drug users stratified by reporting an increase in police presence in the last six months (*n *= 252)

	**Reported an increase in police presence**			
**Characteristic**	**Yes (*n *= 137)**	**No (*n *= 115)**	Odds Ratio (95% CI)*	***P *value**
**Injection heroin use**				
<Daily	88 (64%)	67 (58%)	1.29 (0.77 – 2.14)	0.332
≥Daily	49 (36%)	48 (42%)		
				
**Yaba/Ice use**				
<Daily	107 (78%)	81 (70%)	1.50 (0.85 – 2.65)	0.165
≥Daily	30 (22%)	34 (30%)		
				
**Midazolam use**				
<Daily	97 (71%)	80 (70%)	1.06 (0.62 – 1.82)	0.831
≥Daily	40 (29%)	35 (30%)		
				
**Illicit methadone use**				
<Daily	48 (35%)	36 (31%)	1.18 (0.70 – 2.01)	0.531
≥Daily	89 (65%)	79 (69%)		
				
**Binge drug use**				
<Daily	59 (43%)	40 (35%)	1.42 (0.85 – 2.37)	0.181
≥Daily	78 (57%)	75 (65%)		
				
**Involvement in the sex trade**				
No	17 (12%)	14 (12%)	1.02 (0.48 – 2.18)	0.955
Yes	120 (88%)	101 (18%)		
				
**Involvement in drug treatment**				
No	67 (49%)	49 (43%)	1.29 (0.78 – 2.12)	0.318
Yes	70 (51%)	66 (57%)		

Note: Methadone use refers only to non-prescription (i.e., illicit) use

*CI = Confidence Interval

In this cross-sectional study of drug use patterns among a cohort of Thai IDU, we observed high reported levels of daily injection heroin, midazolam and methamphetamine use, as well as high levels of daily non-injection illicit methadone use. Additionally, although more than half of the cohort participants reported observing an increase in police presence in the six months prior to being interviewed, the time period following the announcement of a renewed drug war, we found no significant bivariate associations between a perceived increase in police presence and a variety of indicators of drug use severity and related risk behaviors among our cohort.

The high levels of injection drug use and polydrug use that we observed among this cohort as well as the apparent negligible impact of an increase in police presence on intensity of drug use or related risk behaviors raise concern given Thailand's continued reliance on an aggressive, and often violent, enforcement-based approach to drug control [9]. The high level of injection midazolam use that we observed among study participants is also of interest. Midazolam is a prescription benzodiazepine and our data suggest that some Thai IDU may be substituting illicit drug use with the misuse of this licit drug. Previous studies have found that a transition or increase in injection drug use as well as an initiation of, or increase in, misuse of licit drugs may occur among drug using populations experiencing an increase in drug enforcement or a decrease in the supply of illicit drugs [8,10,11]. Studies of Thai IDU have also suggested that increases in midazolam injection may have been related to declines in the availability of heroin and subsequent increases in the price of this drug [12]. However, it is notable that heroin injection was widespread among IDU in this cohort. It is also useful to compare the drug use behaviours that we observed among our cohort with results from other studies in our setting. For instance, in a study conducted by Wattana et al. using data from 2004, 19% of a cohort of IDU in Bangkok reported engaging in heroin injection and 2% reported engaging in either injection midazolam or methamphetamine use [13]. These levels are much lower than those we observed in the present study, with 91% of participants reporting injection heroin use, 66% reporting injection midazolam use, and 57% reporting injection methamphetamine use. While these differences may reflect the different sampling strategies used, the higher rates of drug use observed in the present study suggest that drug use has not declined since the first Thai War on Drugs was initiated in 2003. These results also raise questions concerning a possible effect of the Thai government's current response to illicit drug use on the diversification of drug use patterns among this cohort. Further study is of this issue is therefore warranted. Specifically, future studies should attempt to track perceived police presence over time to further assess the impact of enforcement-based policies and practices on drug use and related risks among Thai IDU.

Our study has several limitations. First, our sample was not randomly selected and our findings may not therefore be generalizable to other Thai IDU. Second, due to the cross-sectional study design we caution against inferring a causal association between the independent variables we identified in the present study and a reported increase in police presence among Thai IDU. Specifically, we were unable to compare previous levels of midazolam use among our cohort with the levels reported in this study. As such, we cannot conclude that midazolam use has increased among our sample, though it is noteworthy that the substitution of heroin for midazolam has been observed among other samples of Thai illicit drug users [12]. We were also unable to compare previous levels of arrests with recent levels of arrests among our cohort participants, and we were therefore unable to determine whether arrests increased among Thai IDU upon the implementation of the second Thai War on Drugs, though reports from our study setting suggest that this likely occurred [2]. Further, although more longitudinal research is needed in this area, our study nevertheless reveals high levels of drug use and risk behavior in the presence of an aggressive drug law enforcement campaign. Third, we relied on self-report and socially stigmatized behaviors may therefore have been underreported. Fourth, while we found no significant association between a reported increase in police presence and a number of indicators of drug use or risky drug using behaviors, this may be related to the size of the sample included in this study. However, we note that we have been able to use this sample to detect associations between drug use and HIV risk behaviors (syringe sharing) and drug-related harms (overdose) (data not shown), and we observed no trend towards a significant association between our independent and dependent variables. Regardless, we cannot rule out the possibility of a Type II error.

In summary, we observed high levels of use of diverse types of illicit and licit drugs by both injection and non-injection among a cohort of Thai IDU. We found no association between a reported increase in police presence and a variety of indicators of intensity of drug use and related risk behaviors. These findings, considered alongside reports of extrajudicial killings and other human rights violations accompanying Thailand's drug control strategy, suggest that a reevaluation of the country's reliance on enforcement and violent crackdowns to curtail illicit drug use is urgently needed.

## Declaration of competing interests

The authors declare that they have no competing interests.

## Authors' contributions

DW and TK drafted the original manuscript. KH coordinated data collection. NF, KH, KK and PS offered substantial revisions to the manuscript. All authors read and approved the final manuscript.

## Supplementary Material

Additional file 1 **Table S1. Prevalence and intensity of injection and non-injection drug use among a cohort of Thai injection drug users (*n *= 252)**. Description of drug use levels in the past 6 months among participants in the MSHRC cohort, Bangkok, Thailand. Click here for file

## References

[B1] Ammesty International (2003). Thailand: Grave developments – Killings and other abuses.

[B2] Roberts M, Trace M, Klein A (2004). Thailand's 'War on Drugs'. Drugscope Briefing Papers.

[B3] HRW (2004). Not enough graves.

[B4] Thepkanjana P (2004). Keynote address at the observance of the United Nations International Day Against Drug Abuse and Illicit Trafficking. http://www.un.org/depts/dhl/drug/index.html.

[B5] (2002). Narcotic Addict Rehabilitation Act, B.E. 2545.

[B6] Wong-Anan N (2008). Thai PM vows "rigorous" war on drugs despite outcry. Reuters Bangkok.

[B7] Vongchak T, Kawichai S, Sherman S, Celentano DD, Sirisanthana T, Latkin C, Wiboonnatakul K, Srirak N, Jittiwutikarn J, Aramrattana A (2005). The influence of Thailand's 2003 'war on drugs' policy on self-reported drug use among injection drug users in Chiang Mai, Thailand. Int J Drug Policy.

[B8] Daosodsai P, Bellis MA, Hughes K, Hughes S, Daosodsai S, Syed Q (2007). Thai War on Drugs: Measuring changes in methamphetamine and other substance use by school students through matched cross sectional surveys. Addictive Behaviors.

[B9] HRW (2008). Thailand: New anti-drug campaign risks abuses.

[B10] Small W, Kain S, Laliberte N, Schechter MT, O'Shaughnessy MV, Spittal PM (2005). Incarceration, addiction and harm reduction: inmates experience injecting drugs in prison. Subst Use Misuse.

[B11] Strathdee SA, Zafar T, Brahmbhatt H, Baksh A, ul Hassan S (2003). Rise in needle sharing among injection drug users in Pakistan during the Afghanistan war. Drug & Alcohol Dependence.

[B12] van Griensven F, Pitisuttithum P, Vanichseni S, Wichienkuer P, Tappero JW, Sangkum U, Kitayaporn D, Phasithipol B, Orelind K, Choopanya K (2005). Trends in the injection of midazolam and other drugs and needle sharing among injection drug users enrolled in the AIDSVAX B/E HIV-1 vaccine trial in Bangkok, Thailand. Int J Drug Policy.

[B13] Wattana W, van Griensven F, Rhucharoenpornpanich O, Manopaiboon C, Thienkrua W, Bannatham R, Fox K, Mock PA, Tappero JW, Levine WC (2007). Respondent-driven sampling to assess characteristics and estimate the number of injection drug users in Bangkok, Thailand. Drug Alc Depend.

