# Supplementary material for: Drug use patterns among Thai illicit drug injectors amidst increased police presence
Source: Subst Abuse Treat Prev Policy. 2009 Jul 21;4:16. doi: 10.1186/1747-597X-4-16 (PMC2725037; doi:10.1186/1747-597X-4-16)
Supplement: Additional file 1 — Table S1. Prevalence and intensity of injection and non-injection drug use among a cohort of Thai injection drug users (n = 252). Description of drug use levels in the past 6 months among participants in the MSHRC cohort, Bangkok, Thailand. [file 1747-597X-4-16-S1.pdf]

Table s1. Prevalence and intensity of injection and non-injection drug use among a cohort of Thai injection drug users (*n* = 252)

	Injection (times per day)*				Non-injection (times per day)*			
Type of Drug	Less than daily	1 – 2	> 2 daily	Total	Less than Daily	1 - 2	> 2 Daily	Total
**Heroin**	74 (29.4%)	113 (44.8%)	42 (16.7%)	**229 (90.9%)**	1 (0.4%)	9 (3.6%)	9 (3.6%)	**19 (7.5%)**
**Yaba/Ice**	59 (23.4%)	66 (26.2%)	20 (7.9%)	**143 (56.7%)**	35 (13.9%)	32 (12.7%)	26 (10.3%)	**93 (36.9%)**
**Ecstasy**	--	--	--	**--**	5 (2.0%)	4 (1.6%)	--	**9 (3.6%)**
**Midazolam**	34 (13.5%)	98 (38.9%)	34 (13.5%)	**166 (65.9%)**	1 (0.4%)	7 (2.8%)	2 (0.8%)	**10 (4.0%)**
**Methadone**	3 (1.2%)	9 (3.6%)	2 (0.8%)	**14 (5.6%)**	3 (1.2%)	73 (29.0%)	--	**76 (30.2%)**
**Opiate**[†]	7 (2.8%)	4 (1.6%)	--	**11 (4.4%)**	2 (0.8%)	3 (1.2%)	--	**5 (2.0%)**
**Morphine**	3 (1.2%)	1 (0.4%)	--	**4 (1.6%)**	--	--	--	**--**
**Benzodiazepine**	--	--	--	**--**	6 (2.4%)	14 (5.6%)	10 (4.0%)	**30 (11.9%)**
**Ketamine**	--	--	--	**--**	4 (1.6%)	1 (0.4%)	--	**5 (2.0%)**

Note: Ecstasy, Benzodiazepine and Ketamine are only consumed through non-injection.

**Percentages denote proportion of the total study sample*

†*Opiate refers to opiates other than heroin or methadone*
